# Sleep Disorders in Parkinson’s Disease: Clinical Features, Iron Metabolism and Related Mechanism

**DOI:** 10.1371/journal.pone.0082924

**Published:** 2013-12-23

**Authors:** Shu-yang Yu, Li Sun, Zhuo Liu, Xi-yan Huang, Li-jun Zuo, Chen-jie Cao, Wei Zhang, Xiao-min Wang

**Affiliations:** 1 Department of Neurology, Beijing Tiantan Hospital, Capital Medical University, Beijing, China; 2 Department of Physiology, Capital Medical University, Beijing, China; Hospital General Dr. Manuel Gea González, Mexico

## Abstract

**Objective:**

To investigate clinical features, iron metabolism and neuroinflammation in Parkinson’s disease (PD) patients with sleep disorders (SD).

**Methods:**

211 PD patients were evaluated by Pittsburgh Sleep Quality Index (PSQI) and a body of scales for motor symptoms and non-motor symptoms. 94 blood and 38 cerebral spinal fluid (CSF) samples were collected and iron and its metabolism-relating proteins, neuroinflammatory factors were detected and analyzed.

**Results:**

136 cases (64.5%) of PD patients were accompanied by SD. Factor with the highest score in PSQI was daytime dysfunction. Depression, restless leg syndrome, autonomic symptoms and fatigue contributed 68.6% of the variance of PSQI score. Transferrin level in serum and tumor necrosis factor–α level in CSF decreased, and the levels of iron, transferrin, lactoferrin and prostaglandin E_2_ in CSF increased in PD patients with SD compared with those without SD. In CSF, prostaglandin E_2_ level was positively correlated with the levels of transferrin and lactoferrin, and tumor necrosis factor–α level was negatively correlated with the levels of iron, transferrin and lactoferrin in CSF.

**Conclusions:**

Depression, restless leg syndrome, autonomic disorders and fatigue are the important contributors for the poor sleep in PD patients. Abnormal iron metabolism may cause excessive iron deposition in brain and be related to SD in PD patients through dual potential mechanisms, including neuroinflammation by activating microglia and neurotoxicity by targeting neurons. Hence, inhibition of iron deposition-related neuroinflammation and neurotoxicity may cast a new light for drug development for SD in PD patients.

## Introduction

Sleep disorders (SD) is one of the most common and incapacitating non-motor symptoms in Parkinson’s disease (PD), with an estimated prevalence of 50% [1], and is recognized as an important independent determinant for impaired activity of daily living and quality of life [2]. However, clinical features of SD, particularly the relationship of SD with motor symptoms and other non-motor symptoms are not profoundly recognized yet. Moreover, few investigations focused on the potential etiology and underlying mechanisms of SD in PD patients.

Increasing pathological and autopsy studies on PD patients observed an excessive iron deposition in substantia nigra, which might be caused by iron metabolism dysfunction due to gene mutations involving iron metabolism-relating proteins [3] or excessive iron uptake [4]. Deposited iron in substantia nigra of PD brain was demonstrated to induce progressive dopaminergic neurodegeneration, and propagate motor symptoms through neuroinflammatory mechanism [5]. Neuroinflammatory mechanism characterized by microglia activation plays an important role in PD,leading to progressive degeneration and death of dopaminergic neurons in substantia nigra. However, what role it plays in SD of PD is not clear yet. The questions raised here are whether iron deposition is a potential etiology of PD-SD, and whether neuroinflammation is the underlying mechanism of PD-SD? These issues almost haven’t been studied before and remain enigmas yet.

In this study, we investigated the clinical features, relevant factors of SD in PD patients. The levels of iron and its metabolism-relating proteins, and neuroinflammatory factors in both serum and cerebrospinal fluid (CSF) in PD patients with and with no SD were detected and analyzed.

## Methods

### Subjects

211 PD patients were consecutively recruited from the Department of Neurology, Beijing Tiantan Hospital, Capital Medical University from April 2010 to April 2012. PD was diagnosed according to UK Parkinson’s Disease Society Brain Bank [6].

Among 211 PD patients, 112 cases (53.1%) were male and 90 cases (46.9%) were female; patients’ age were 35–85 years with an average of 60.2 years; disease duration was from one month to 26 years with an average of 2.0 years. All PD patients were evaluated by Pittsburgh Sleep Quality Index (PSQI), and recruited in SD group (with PSQI≥5 points) and NSD (with no SD) groups (PSQI<5 points), respectively.

### Ethics Statement

This study was approved by Beijing Tiantan Hospital review board. Written informed consents were obtained from all participants in this study.

### Assessments of Clinical Features

#### Motor symptoms and motor complications

Motor symptoms in SD and NSD groups was evaluated by the Unified Parkinson’s Disease Rating Scale III, Hohen and Yahr Stage and Modified Webster Scale. Motor complications were assessed by Unified Parkinson’s Disease Rating Scale IV and Wearing –Off Scale.

#### Non-motor symptoms

Non-motor symptoms were screened by Non-motor Symptoms Quest followed by series of scales, including Montreal Cognitive Assessment and Mini-Mental State Examination for cognitive impairment, Hamilton Depression Scale -24 items for depression, Hamilton Anxiety Scale -14 items for anxiety, The Scale For Outcomes in PD For Autonomic Symptoms for autonomic dysfunction, Restless Leg Syndrome Rating Scale for restless leg symptom and Fatigue Sale-14 items for fatigue.

### Sample Collections

#### Blood sample collection

Anti-parkinsonian drugs were stopped for 12–14 hours if patients’ condition allowed. 3 ml blood was taken under fasting condition, followed by being centrifuged in 4°C at 3000 r/min for 10 minutes. Supernatant was preserved at −80°C for detection.

#### Cerebrospinal fluid sample collection

Procedure was the same as 3.1 blood sample collection except that 3 ml cerebrospinal fluid was collected through lumbar puncture.

### Detection of Iron and its Metabolism-relating Proteins

Iron and its metabolism-relating proteins, including transferrin, lactoferrin and ferritin in serum and CSF from PD patients were detected by Enzyme Linked Immunosorbent Assay. Ab83366 kit for iron, Ab108837 kit for ferritin and Ab108911 kit for transferrin were all from Abcam Company (British). E01L0224 kit for lactoferrin was from Shanghai Lanji Biological Limited Company (China).

### Detection of Neuroinflammatory Factors

#### Tumor necrosis factor-α, interleukin-1β and prostaglandin E_2_

Tumor necrosis factor-α, interleukin-1β and prostaglandin E_2_ in serum and CSF from PD patients were detected by enzyme linked immunosorbent assay. 1R350 kit for tumor necrosis factor-α and 1R040 kit for interleukin-1β were both from Rapidbio Company (USA). CSB-E07965h kit for prostaglandin E_2_ was from Cusabio Company (China).

#### Hydrogen peroxide

Hydrogen peroxide in serum and CSF from PD patients were detected by chemical colorimetric method. A064 kit was from Nanjing Jiancheng Biological Engineering Research Institute (China).

#### Nitric oxide

Nitric oxide in serum and CSF from PD patients were detected by chemical colorimetric method. A012 kit was from Nanjing Jiancheng Biological Engineering Research Institute (China).

### Data Analyses

Statistical analyses were performed with SPSS Statistics 20.0 (IBM Corporation, New York, USA). Demographic information, disease features, motor symptoms, motor complications, non-motor symptoms, biochemical factors in serum and CSF were compared between SD and NSD groups. Multiple linear analyses were performed between SD and related factors. Spearman correlation analyses were made among biochemical factors. Continuous variables were presented as means ± standard deviations and compared by 2-tailed T test if they were normally distributed, and presented as median (quartile) and compared by nonparametric test if they were not normally distributed. Discrete variables were compared by Chi square test. Bonferroni correction was performed in the assessment of clinical symptoms. We selected 12 factors that might affect sleep as independent variables (sex, age, age of onset, educational level, disease duration, onset lateralization, clinical phenotype, motor symptoms, motor complications, autonomic dysfunction, abnormal sensory disorders and neuropsychiatric symptoms), and found that the corrected P value was significant when it was less than 0.004. In the detection of biochemical factors, P value was significant when it was less than 0.05.

## Results

### Incidence of SD in PD Patients

136 of 211 (64.5%) PD patients had SD, among them, 32 cases(15.2%)had SD before motor symptoms.

### Assessment of Sleep Status of PD Patients

The average scores of PSQI and its seven factors were compared between SD and NSD groups (**Table 1**). Daytime dysfunction ranked the top of PSQI, followed by sleep disturbances and sleep latency. Seven factors of PSQI were all significantly impaired in SD group.

**Table 1:** Pittsburgh Sleep Quality Index and its seven factors in SD group and NSD group.

Pittsburgh Sleep Quality Index	Total PD	SD group	NSD group	P value
	(Mean)	[Median(quartile)]	[Median(quartile)]	
Sleep quality	1.2	1.0 (1.0∼2.0)	1.0 (0.0∼1.0)	0.000
Sleep latency	1.2	2.0 (1.0∼3.0)	0.0 (0.0∼1.0)	0.000
Sleep duration	0.8	1.0 (0.0∼2.0)	0.0 (0.0∼0.0)	0.000
Habitual sleep efficiency	0.8	1.0 (0.0∼2.0)	0.0 (0.0∼0.0)	0.000
Sleep disturbances	1.3	1.0 (1.0∼2.0)	1.0 (1.0∼1.0)	0.000
Use of sleeping medication	0.4	0.0 (0.0∼0.8)	0.0 (0.0∼0.0)	0.000
Daytime dysfunction	1.6	2.0 (1.0∼3.0)	1.0 (0.0∼1.0)	0.000

SD: sleep disorders; NSD: no sleep disorders.

### Comparisons of Demographics Information, Disease Features and Non-motor Symptoms between SD and NSD Groups

Demographics information, disease features and non-motor symptoms were compared between SD and NSD groups (**Table 2** **,** **3**). Results showed that SD group had significantly more severe motor symptoms and more non-motor symptoms than NSD group. The incidences of psychiatric symptoms (loss of interest, difficulty concentrating or staying focused, feeling sad, feeling anxious, frightened or panicky, feeling less interested in sex or more interested in sex) and abnormal sensory (unexplained pains) were increased in NSD group.

**Table 2:** Demographics information, disease features in SD group and NSD group.

	SD group	NSD group	P value
Female/Total [case/total (%)]	72/136 (52.9%)	40/75 (53.3%)	NS
Age [median (quartile)]	60.0 (4.0∼67.0)	60.0 (52.3∼68.0)	NS
Educational level			NS
Primary school and below [case/total (%)]	52/136 (38.2%)	20/75 (26.7%)	
Middle and high school [case/total (%)]	65/136 (47.8%)	34/75 (45.3%)	
Bachelor and above [case/total (%)]	19/136 (14.0%)	21/75 (28.0%)	
Age of onset (years, mean ± SD)	56.6±9.8	56.5±11.1	NS
Disease duration [median (quartile)]	2.0 (1.0∼4.5)	3.0(1.0∼5.8)	NS
Onset lateralization			NS
Lest side [case/total (%)]	60/123 (48.8%)	29/66 (43.9%)	
Right side [case/total (%)]	55/123 (44.7%)	30/66 (45.5%)	
Both sides [case/total (%)]	8/123 (6.5%)	7/66 (10.6%)	
Clinical phenotype			NS
Tremor type [case/total (%)]	10/99 (10.1%)	6/46 (13.0%)	
Rigidity-bradykinesia type [case/total (%)]	20/99 (20.2%)	4/46 (8.7%)	
Mixed type [case/total (%)]	69/99 (69.7%)	36/46 (78.3%)	
Unified Parkinson’s Disease Rating Scale III [median (quartile)]	26.0(18.0∼35.0)	21.0(13.5∼30.0)	0.001
Unified Parkinson’s Disease Rating Scale IV [median (quartile)]	0(0∼3.0)	0(0∼2.0)	NS
Numbers of non-motor symptoms [median (quartile)]	13.0(10.0∼16.8)	7.0(4.0∼10.0)	0.000

SD: sleep disorders; NSD: no sleep disorders; NS: not significant, P>0.004.

**Table 3:** Incidences of non-motor symptoms in SD group and NSD group [case/total (%)].

Non-motor symptoms	SD group	NSD group	P value
Dribbling of saliva during the daytime	60/133 (45.1%)	19/74 (25.7%)	NS
Loss or change in the ability to taste or smell	67/133 (50.4%)	22/74 (29.7%)	NS
Swallowing difficulty or problems with chocking	49/133 (36.8%)	17/74 (23.0%)	NS
Vomiting or feeling of sickness (nausea)	61/133 (45.9%)	22/74 (29.7%)	NS
Constipation	75/133 (56.4%)	32/74 (43.2%)	NS
Bowel incontinence	7/133 (5.3%)	2/74 (2.7%)	NS
Bowel emptying incomplete	33/133 (24.8%)	12/74 (16.2%)	NS
Urine urgency	71/133 (53.4%)	25/74 (33.8%)	NS
Getting up early at night to pass urine	74/133 (55.6%)	29/74 (39.2%)	NS
Unexplained pains	66/133 (49.6%)	18/74 (24.3%)	0.000
Change in weight (not due to change in diet)	52/133 (39.1%)	20/74(27.0%)	NS
Problems remembering things	105/133 (78.9%)	49/74 (66.2%)	NS
Loss of interest	80/133 (60.2%)	23/74 (31.1%)	0.000
Seeing or hearing things that are not there	15/133 (11.3%)	3/74 (4.1%)	NS
Difficulty concentrating or staying focused	72/133 (54.1%)	23/74 (31.1%)	0.001
Feeling sad, ‘low’ or ‘blue’	104/133 (78.2%)	34/74 (45.9%)	0.000
Feeling anxious, frightened or panicky	88/133 (66.2%)	31/74 (41.9%)	0.001
Feeling less interested in sex or more interested in sex	78/133 (58.6%)	27/74 (36.5%)	0.002
Finding it difficult to have sex	75/133 (56.4%)	29/74 (39.2%)	NS
Postural hypotension	47/133 (35.3%)	22/74 (29.7%)	NS
Falling	23/133 (17.3%)	7/74 (9.5%)	NS
Finding it difficult to stay awake	82/133 (61.7%)	12/74 (16.2%)	0.000
Difficulty getting to sleep or staying asleep at night	68/133 (51.1%)	7/74 (9.5%)	0.000
Intense, vivid dreams or frightening dreams	54/132 (40.9%)	10/74 (13.5%)	0.000
Talking or moving about in sleep	36/133 (27.1%)	5/74 (6.8%)	0.000
Unpleasant sensations in legs that you need to move	72/133 (54.1%)	30/74 (40.5%)	NS
Swelling of legs	33/133 (24.8%)	10/74 (13.5%)	NS
Excessive sweating	62/133 (46.6%)	25/74 (33.8%)	NS
Double vision	32/133 (24.1%)	12/74 (16.2%)	NS
Delusion	3/133 (2.3%)	2/74 (2.7%)	NS

SD: sleep disorders; NSD: no sleep disorders; NS: not significant, P>0.004.

### Related Factors of SD in PD Patients

Multiple linear regression analysis was made between SD (PSQI points) and related factors (age, educational level, age of onset, disease duration, numbers of non-motor symptoms, scores of scales for motor symptoms, motor complications and non-motor symptoms) (**Table 4**). Results revealed that depression, restless leg symptom,autonomic dysfunction and fatigue significantly influenced PSQI score, which explained 68.6% variance of total PSQI score.

**Table 4:** Multiple linear regression analysis between SD and related factors.

Factors related to Pittsburgh Sleep Quality Index	β	*P*	△R^2^	Adjusted R^2^
Hamilton Depression Scale	0.227	0.000	0.467	0.453
Restless Leg Syndrome Rating Scale	0.205	0.001	0.593	0.571
The Scale For Outcomes in PD For Autonomic Symptoms	0.223	0.002	0.649	0.620
Fatigue Sale-14 items	−0.489	0.006	0.718	0.686

SD: sleep disorders.

### Levels of Iron and its Metabolism-relating Proteins

The levels of iron, transferrin, lactoferrin and ferrtin in serum and CSF were compared between SD and NSD groups (**Table 5**). Data showed that transferrin level in serum decreased and the levels of iron, transferrin and lactoferrin in CSF increased in SD group.

**Table 5:** Levels of iron and its metabolism-relating proteins, and neuroinflammatory factors in serum and cerebral spinal fluid in SD group and NSD group.

Serum	NSD group (64 cases)	SD group (31 cases)	P value
Iron (nmol)	2.988±1.537	3.221±1.460	NS
Transferrin (ug/ml)	1.590 (0.124∼4.761)	0.095 (0.065∼4.450)	0.041
Lactoferrin (ug/ml)	61.430 (51.594∼88.021)	62.384 (45.202∼90.399)	NS
Ferrtin (ng/ml)	8.951 (6.143∼13.855)	9.223 (6.646∼14.068)	NS
Tumor necrosis factor-α (pg/ml)	63.317 (49.430∼68.023)	64.884 (52.813∼74.446)	NS
Prostaglandin E_2_ (pg/ml)	8.145 (4.457∼14.021)	5.619 (3.763∼10.770)	NS
Interleukin-1β(pg/ml)	8.480 (5.471∼74.171)	12.042 (6.154∼165.780)	NS
Hydrogen peroxide (mmol/l)	28.882 (23.938∼35.316)	28.025 (24.593∼30.312)	NS
Nitric oxide (mmol/l)	44.851±28.426	52.784±29.106	NS
**Cerebral spinal fluid**	**NSD group (12 cases)**	**SD group (26 cases)**	**P value**
Iron (nmol)	0.357 (0.280∼0. 393)	0.496 (0.322∼0.579)	0.004
Transferrin (ug/ml)	0.152 (0.139∼0.281)	0.301(0.154∼0.363)	0.041
Lactoferrin (ug/ml)	118.885±46.098	171.359±45.357	0.002
Ferrtin (ng/ml)	3.766 (3.091∼12.667)	11.756 (2.356∼16.023)	NS
Tumor necrosis factor-α(pg/ml)	515.147(482.967∼636.290)	45.700 (32.809∼479.859)	0.001
Prostaglandin E2 (pg/ml)	5.142 (4.643∼5.767)	6.074 (4.363∼11.273)	0.022
Interleukin-1β(pg/ml)	9.026 (6.296∼76.186)	27.702 (8.576∼165.393)	NS
Hydrogen peroxide (mmol/l)	1.973 (1.349∼2.368)	1.973 (1.432∼2.106)	NS
Nitric oxide (mmol/l)	56.705±20.705	53.793±28.746	NS

SD: sleep disorders; NSD: no sleep disorders; NS: not significant, P>0.05.

### Levels of Neuroinflammatory Factors and their Correlations with the Levels of Iron and its Metabolism-relating Proteins

The levels of neuroinflammatory factors, including tumor necrosis factor-α, prostaglandin E_2_, interleukin-1β, hydrogen peroxide and nitric oxide in serum and CSF were compared between SD and NSD groups (**Table 5**). An elevated PGE_2_ level in CSF was found in SD group compared with NSD group. Further analyses demonstrated positive correlations between the levels of prostaglandin E_2_ and transferrin (r = 0.335, P = 0.009), prostaglandin E_2_ and lactoferrin (r = 0.290, P = 0.026) in CSF. A declined tumor necrosis factor-α level in CSF was found in SD group compared with NSD group. Subsequent analyses indicated negative correlations between the levels of tumor necrosis factor-α and iron (r = −0.421, P = 0.001), tumor necrosis factor-α and transferrin (r = −0.777, P = 0.000), tumor necrosis factor-α and lactoferrin (r = −0.509, P = 0.000) in CSF.

## Discussion

In this investigation, SD incidence reached to 64.5%, demonstrating that SD was a very common non-motor symptom in PD patients. The scores of seven factors of PSQI in SD group were significantly higher than that in NSD group (**Table 1**), illustrating that all these aspects were dramatically impaired. Daytime dysfunction, ranking the top of PSQI, might be related to poor nocturnal sleep, disease progression and daily L-dopa dose [7]. Sleep disturbances ranked the second place, affecting 74%–88% PD patients [8]. Sleep disturbances can be induced by impaired thalamocortical arousal system, degeneration of sleep-wakefulness regulatory centers in brainstem, motor symptoms, non-motor symptoms and dopaminergic medication, which may coexist and amplify the effect of each other during SD progression [9]. Sleep latency was in the third place, indicating less impairment in falling sleep than sleep disturbances which was supported by other studies [10]. Sleep duration and habitual sleep efficiency also declined in SD group. Sleep quality was barely acceptable and poor in PD patients with SD. The frequency of taking sleep pills in PD patients in this study was less than once a week, indicating an insufficient therapy for SD in PD patients.

Comparison of demographic information displayed no significant difference in sex, age, age of onset and educational level (**Table 2**) between SD and NSD groups, demonstrating that demographic information was not related to PD-SD. Onset lateralization, disease duration and clinical phenotype were not different between SD and NSD groups**,** consistent with the previously published data [11], [12]. It was motor symptoms evaluated by UPDRS III score but not motor complications assessed by UPDRS IV score impaired the sleep strikingly (**Table 2**).

We found more non-motor symptoms in SD group than NSD group (**Table 2**). The incidences of non-motor symptoms, such as psychiatric symptoms and abnormal sensory in SD group were all significantly higher than NSD group (**Table 3**). However, the incidence of autonomic dysfunction, was not significantly different between the two groups, which might be because that autonomic dysfunction presented earlier than SD according to Braak stage [13]. Other symptoms showed no significant difference between the two groups, e.g. hallucination, delusion, falling, diplopia and body weight change, etc, which are generally common in the later stage of PD and occur after SD, and thus they might not play causative role in PD-SD. Multiple linear regressions analysis indicated that non-motor symptoms were more pivotal than motor symptoms or motor complications for PD-SD (Table 4).

Pathology and autopsy studies observed excessive iron deposition in substantia nigra, with total iron elevated by 25% to 100% and iron ions by 225% [14], [15]. Peripheral iron is transferred into brain through blood-brain barrier. In brain, capillary endothelial cells absorb iron-bound transferrin from blood via receptor-mediated endocytosis. Iron then is released from endosome into interstitial fluid by iron exporter ferroportin [16]. Iron in brain interstitium may bind to large molecules, such as transferrin or lactoferrin, and then is transported into neurons by related receptors. Excessive intake of exogenous iron [17], gene mutation of iron metabolism-relating proteins [3] and blood-brain barrier impairment [18] may induce redundant iron deposition in the brain. Studies on iron and PD-SD patients mainly focused on restless leg symptom [19], [20],and found that iron deposition in substantia nigra was negatively correlated with restless leg symptom. Presently, no exploration has been conducted between iron metabolism and PD-SD assessed by PSQI.

Here, we found that iron level in CSF in PD-SD group was dramatically elevated compared with PD-NSD group (Table 5), implying an excessive iron deposition in brain. The level of lactoferrin in CSF in PD-SD group was obviously enhanced compared with PD-NSD group, suggesting an abnormal iron metabolism in brain. Iron level in serum in PD-SD group wasn’t different with PD-NSD group, indicating that elevated iron in brain wasn’t resulted from iron elevation in the peripheral system. Transferrin level in serum was significantly decreased, reflecting that the enhanced brain iron might due to the translocation of transferrin from peripheral system to central system, a process that highly contributed to iron accumulation in brain. Hence, abnormal iron metabolism in brain might cause iron overload and be related to PD-SD.

Mesocorticolimbic dopamine system, a part of thalamocortical arousal system, which is originated from ventral tegmental area and innervates to thalamus and hippocampus [21], [22]. Dysfunction of mesocorticolimbic dopamine system is featured by a lack of normal rhythms during night time and an excessive sleepiness during daytime [23], indicating a key role of dopamine system in sleep control, specifically in wakefulness maintainance and sleep regulation. Iron often deposit in the areas containing the cells enriched in dopamine, such as substantia nigra, and the fibers innervated from dopaminergic neurons, such as mesocorticolimbic dopamine system [21], which might be relevant to PD-SD. Moreover, Lewy bodies were observed in the sleep-regulating centers [21], including locus coeruleus, raphe dorsal, laterodorsal tegmental nucleus/pedunculopontine tegmental nucleus, tuberomammillary nucleus and ventrolateral preoptic nucleus [24],with PD pathology progression [25] and meanwhile, iron was found inside Lewy bodies in above sleep-regulating centers [26], which implied that iron might be relevant to PD-SD. Therefore, inhibition of iron deposition might serve as a target for PD-SD therapy.

Compared with PD-NSD group, prostaglandin E_2_ level in PD-SD group was significantly elevated in CSF but not in serum, indicating a potential role of neuroinflammation in the brain of PD patient with SD. A study revealed that prostaglandin E_2_ was elicited by insufficient sleep [27]. Elevated prostaglandin E_2_ might propagate the deterioration of PD-SD, which might induce further release of PGE_2_, forming a vicious cycle for PD-SD progression.

Tumor necrosis factor-α level in CSF in PD-SD group was strikingly declined compared with PD-NSD group. However, tumor necrosis factor-α level in serum in PD-SD group wasn’t prominently different with PD-NSD group. It was found that tumor necrosis factor-α was linked to sleep promotion [24]. Administration of exogenous tumor necrosis factor-α increased slow wave sleeps and inhibition of tumor necrosis factor-α significantly reduced sleep amount [24]. Thus, the decreased tumor necrosis factor-α level in CSF was relevant to PD-SD due to its less sleep-promoting effect.

Although interleukin-1β was thought to be linked to sleep [24], we observed no differences in the levels of interleukin-1β as well as nitric oxide and hydrogen peroxide in both serum and CSF between SD and NSD groups, indicating that they were not the potential indicators for PD-SD.

Further analysis showed significant positive correlations between the levels of prostaglandin E_2_ and transferrin (r = 0.335, P = 0.009), and the levels of prostaglandin E_2_ and lactoferrin (r = 0.290, P = 0.026) in CSF, indicating iron overload might be relevant to PD-SD by inducing neuroinflammation [28], [29]. Additionally, we found negative correlations between the levels of tumor necrosis factor-α and iron (r = −0.421, P = 0.001), tumor necrosis factor-α and transferrin (r = −0.777, P = 0.000), tumor necrosis factor-α and lactoferrin (r = −0.509, P = 0.000) in CSF. Although tumor necrosis factor-α was produced by catalyzing intracellular reactive oxygen species in alveolar macrophages [[28], [29], we failed to find that iron increased the level of tumor necrosis factor-α, suggesting that iron might cause direct neurotoxicity instead of activating microglia and producing tumor necrosis factor-α. Neuroinflammatory factors detected here showed different patterns of production, which reasons remain unclear yet and need to be figured out in the near future.

In summary, PD patients have high SD incidences. Depression, restless leg symptom, autonomic dysfunction and fatigue are the main contributors for PD-SD. we for the first time find that abnormal iron metabolism may cause iron deposition in brain and be related to PD-SD through dual mechanisms of neuroinflammation by activating microglia and neurotoxicity by targeting neurons. Hence, inhibition of excessive iron deposition-induced neuroinflammation [30], [31] and neurotoxicity may cast a new light for drug development for PD-SD.
